# Supplementary figures and images for: Roles for the RNA-Binding Protein Caper in Reproductive Output in Drosophila melanogaster
Source: J Dev Biol. 2022 Dec 23;11(1):2. doi: 10.3390/jdb11010002 (PMC9844462; doi:10.3390/jdb11010002)

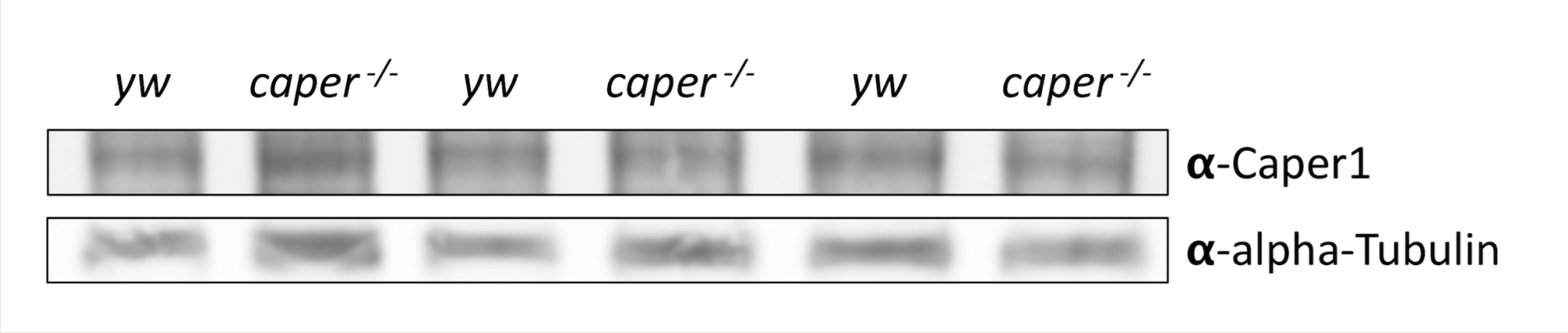

Supplement: Supplementary file 1 [file jdb-11-00002-s001.zip › jdb-1854598-supplementary.png]
